# Prevalence of nonrestorative sleep before and during the COVID-19 pandemic: based on a nationwide cross-sectional survey among Japanese in 2019 and 2022

**DOI:** 10.1265/ehpm.24-00197

**Published:** 2025-01-25

**Authors:** Kimiko Tomioka, Midori Shima, Keigo Saeki

**Affiliations:** Nara Prefectural Health Research Center, Nara Medical University, Kashihara, Nara, Japan

**Keywords:** Nonrestorative sleep, Sleep duration, COVID-19, National representative survey, Cross-sectional study, Japan

## Abstract

**Background:**

Japanese people sleep less compared to other countries around the world. Using a large nationally representative survey in 2019 and 2022, we investigated whether sleep duration and nonrestorative sleep (NRS) among Japanese people have improved or worsened due to the COVID-19 pandemic.

**Methods:**

Data were drawn from the Comprehensive Survey of Living Conditions, a nationwide cross-sectional sample based on self-administered questionnaires. We analyzed 426,510 people in 2019 and 375,578 people in 2022 aged ≥20 living in the community. The generalized estimating equations of the multivariable Poisson regression models were used to estimate adjusted prevalence of NRS by survey year. Potential confounders included gender, age, marital status, family size, housing tenure, equivalent household expenditures, education, employment status, illness under treatment, lifestyle behaviors (i.e., smoking, drinking, dietary, and fitness habits), mental health, and sleep duration.

**Results:**

Among the study participants, 35.7% slept less than 6 hours and 20.9% had NRS. Regarding sleep duration, the prevalence of sleep duration of less than 6 hours was significantly lower in 2022 than in 2019 for both men and women. By gender and age, the prevalence of short sleep duration (<6 hours) significantly decreased for both men and women under the age of 49, but increased significantly for men aged ≥50 and women aged ≥75. Regarding NRS, the prevalence of NRS was significantly lower in 2022 than in 2019 regardless of gender and age: Prevalence among men was 21.4% in 2019 and 18.8% in 2022, and prevalence among women was 23.7% in 2019 and 21.2% in 2022. After adjustment for potential confounders, the difference between the 2022 NRS prevalence and the 2019 NRS prevalence was minus 1.64 percent point (pp) (95% confidence interval minus 1.82 pp to minus 1.46 pp, *P* < 0.001), showing a significant decrease in the 2022 NRS prevalence. A significant improvement of NRS was independent of the prevalence of short sleep duration, age, gender, and employment status.

**Conclusions:**

The prevalence of NRS among the general population in Japan was significantly reduced during the COVID-19 pandemic compared to before the COVID-19 pandemic. We need to monitor whether this decline continues or returns to pre-pandemic levels.

**Supplementary information:**

The online version contains supplementary material available at https://doi.org/10.1265/ehpm.24-00197.

## Graphical Abstract

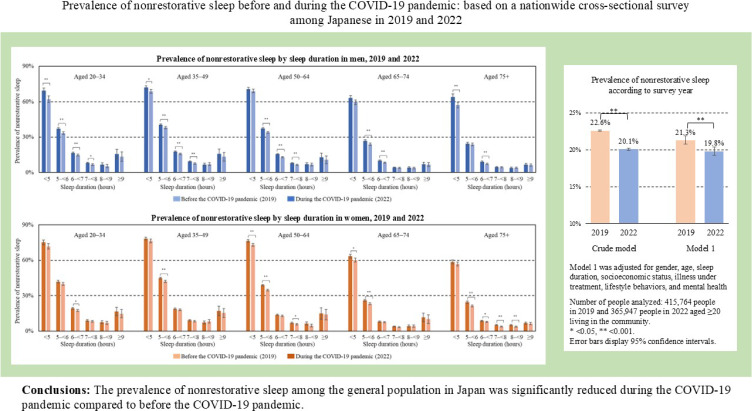

## Background

According to a 2021 survey by the OECD, the average sleep duration of Japanese people was 7 hours and 22 minutes, the shortest among 33 countries [1]. Short sleep duration is a risk factor for developing poor health outcomes such as all-cause mortality, cardiovascular disease, osteoporosis, stroke, and type 2 diabetes [2].

The feeling of being refreshed following sleep has been used in previous research as an indicator of appropriate sleep, that is, subjective sleep quality [3]. Nonrestorative sleep (NRS), defined as a subjective feeling of being insufficiently rested upon waking, is a distinct component of insomnia, but it can occur with or without difficulties initiating and maintaining sleep [4]. It has also been shown that a significant proportion of the population may experience a lack of refreshment upon waking that cannot be explained by lack of sleep [5], and that complaints of NRS can occur even in people with normal sleep onset, duration, and continuity as determined by polysomnography [6]. Japanese cohort studies have reported that NRS is associated with the onset of type 2 diabetes [7], the development of metabolic syndrome [8], and subsequent risk of cardiovascular disease [9]. Against the background of the increasing trend of NRS among Japanese people, “Health Japan 21”, a national health promotion campaign, aims to reduce the proportion of people with NRS [10].

Faced with the COVID-19 pandemic in 2020, various countries implemented movement restrictions and social distancing measures. Although these restrictions were effective in preventing the spread of infection, it has been reported that they affected people’s sleep: A Brazilian online survey found that during the COVID-19 isolation period, interruptions in daily physical activity had a negative impact on sleep quality [11]. It has been reported that deterioration in sleep quality during the COVID-19 pandemic is associated with social isolation due to lockdowns, increased economic stress, reduced sunlight exposure and physical activity due to reluctance to go out, nocturnal lifestyles due to increased working from home and online classes, changes in lifestyle, and direct exposure to COVID-19 [12, 13]. A few studies outside of Japan have reported on the impact of the COVID-19 pandemic on NRS [14, 15], but the study participants have been limited to nursing professionals [14] or older people [15]. These limitations would suggest that it is important to investigate changes in sleep symptoms among Japanese people during the COVID-19 pandemic.

Therefore, this study aimed to examine whether the NRS of Japanese people has improved or worsened due to the COVID-19 pandemic, using data from the previous survey (2019) and the latest survey (2022) of a large cross-sectional Japanese population survey.

## Methods

### Data sources

#### Comprehensive Survey of Living Conditions (CSLC)

The CSLC is a nationally representative cross-sectional survey, conducted by the Ministry of Health, Labour and Welfare in Japan. Details on the CSLC are available elsewhere [16]. Briefly, the CSLC targets households and household members nationwide and is a random sampling method based on households. A large-scale survey (surveying household composition and health status, etc.) is conducted every three years. Specifically, the data is based on self-administered questionnaires from approximately 500,000 individuals, and includes many items such as sleep duration, mental health, lifestyle habits (smoking, drinking, exercise habits), basic attributes (sex, age, family structure, history of current illness), and socioeconomic status (educational background, household expenditure, occupation, employment type) in addition to NRS. Using the CSLC, it is possible to determine the proportion of people with NRS by gender and age, adjusting for important confounding factors.

#### National Database of Health Insurance Claims and Specific Health Checkups of Japan (NDB) Open Data Japan

The NDB Open Data Japan (NDBODJ) has been made available online by the Ministry of Health, Labour and Welfare [17]. The NDBODJ collects data on people who undergo specific health checkups (SHC) nationwide. The target age group for SHC is between 40 and 74 years old. In 2019, the number of people eligible for SHC was 53,798,756, and the number of people who received SHC was 29,774,873, so the proportion of persons receiving SHC was 55.3%. Similarly, for 2022, the number of eligible people was 51,924,629, the number of people receiving SHC was 30,016,491, and the proportion of people receiving SHC was 57.8% [18]. Because NRS is included in the standard questionnaire for SHC, valid NRS data for approximately 27 million people has been available through the NDBODJ. Since the summary tables of the standard questionnaire have been made public every year since the second release, we used the NDBODJ from the second release to the latest ninth release, to calculate the crude prevalence of NRS from 2014 to 2021, and clarify the secular trends in the NRS among Japanese people. The NDBODJ is one of the largest in the world and is highly reliable and useful for evaluating national health issues. However, although the NDBODJ is stratified by gender and age, it cannot adjust for other factors.

### Participants

All data were derived from the CSLC, where questionnaires were distributed to approximately 300,000 randomly selected households each survey year [16]. The proportion of the number of households that gave valid responses to the total number of households receiving the survey was 72.1% in 2019 and 68.0% in 2022, and the number of people with valid responses was 535,619 in 2019 and 472,042 in 2022. Because the CSLC is a survey based on a random sampling method on households nationwide, it may be assumed that the number of household members surveyed corresponds to the population of Japan by gender and age. Based on this assumption, we calculated an estimate of the number of household members to be surveyed and estimated the valid response rate by gender and age group. The results are shown in Additional file 1. Compared with the 2019 survey, those who did not respond to the 2022 survey were estimated to be aged 64 or younger, both men and women. Excluding those under the age of 20, hospitalized people, those with nursing home admission, and whose age or hospitalization/admission status was unknown, we analyzed 426,510 people in 2019 and 375,578 people in 2022 (Additional file 2). The reasons for excluding certain participants from the study are provided in Additional file 3.

### Measures

#### Nonrestorative sleep (NRS)

The presence of NRS was assessed using the following single question: “Have you been well rested through sleep over the past month?” For this question, participants selected one of the following four options: “Very restful”, “restful”, “restless”, and “very restless”. Those who answered either “restless” or “very restless” were defined as those with NRS. The validity of this single self-report question using a four-point Likert scale has not been tested.

#### Sleep duration

Sleep duration was assessed using the following question: “How many hours did you sleep on average per day over the past month?” For this question, participants selected one of six options: less than 5 hours, 5 hours or more but less than 6 hours, 6 hours or more but less than 7 hours, 7 hours or more but less than 8 hours, 8 hours or more but less than 9 hours, and 9 hours or more. Those who answered “less than 5 hours” or “5 hours or more but less than 6 hours” were defined as having short sleep duration.

#### Other variables

Demographic characteristics included gender (men or women) and age group in years (20–34, 35–49, 50–64, 65–74, or ≥75). Socioeconomic status (SES) included marital status (married, never-married, or widowed/divorced), family size (five or more, four, three, two, or living alone), housing tenure (owner-occupied, privately rented, provided housing, publicly subsidized, or rented rooms), equivalent household expenditures (EHE), education (years of schooling) (≤9, 10–12, 13–15, or ≥16), and employment status. Illness under treatment was dichotomized into present or absent. Lifestyle behaviors included smoking status (cigarettes per day) (never-smokers, ex-smokers, current smoking 1–10, current smoking 11–20, or current smoking >20), alcohol intake (average amount of alcohol per day) (non-drinkers, social drinkers, <10 g, 10–39 g, 40–59 g, or ≥60 g), and the number of dietary and fitness habits practiced. Mental health was assessed using the Japanese version of the Kessler 6-item Psychological Distress Scale (K6) [19], and categorized as having serious psychological distress (the K6 score of 13–24), moderate psychological distress (5–12), or no mental health problems (0–4). We have presented a detailed description of age, EHE, employment status, and illness under treatment in Additional file 4. Additionally, the distribution of responses for variables used as covariates is also provided in Additional file 5.

We ensured that all variables were free of multicollinearity problems (i.e., no variable had a variance inflation factor greater than 2.0).

### Statistical analyses

Prevalence between 2019 and 2022 was compared using the Chi-squared test. To estimate adjusted prevalence of NRS by survey year, we used the generalized estimating equations of the multivariable Poisson regression models (Model 1: adjusted for gender, age, and sleep duration; Model 2: Model 1 plus adjusted for socioeconomic status, illness under treatment, lifestyle behaviors, and mental health). The difference in the prevalence of NRS was calculated by subtracting the 2019 prevalence from the 2022 prevalence, and the unit of the NRS prevalence difference was percent point (pp). Statistical analyses were performed using the IBM SPSS Statistics Ver. 27 for Windows (Armonk, New York, NY, United States). Due to the large sample size, for comparisons of crude proportions, statistical significance was set at the *P* < 0.01 level, with effect sizes reported.

## Results

Of the 802,088 study participants, the mean age (standard deviation) was 56.8 (18.2) years, the proportion of men was 47.6%, the prevalence of short sleep duration (<6 hours) was 35.7%, and the prevalence of NRS was 20.9%. The lifestyle behaviors that were significantly more favorable in 2022 than in 2019 for both men and women were current smoking, binge drinking, eating meals regularly, eating a balanced diet, and doing regular exercise. Regarding mental health, there was no significant difference between 2019 and 2022 for both men and women (Table 1).

**Table 1:** Characteristics of lifestyle behaviors and mental health among study participants by gender and survey year

	**Men**								**Women**							
	**Before (2019)**			**During (2022)**			***P***	***ES*** ***φ***	**Before (2019)**			**During (2022)**			***P***	***ES*** ***φ***
	**Absent**	**Present**	**% of present**	**Absent**	**Present**	**% of present**			**Absent**	**Present**	**% of present**	**Absent**	**Present**	**% of present**		
Smoking habits																
Current smoking	139,396	59,045	29.8	128,789	46,729	26.6	<0.001	-0.035	201,106	18,471	8.41	179,435	14,565	7.51	<0.001	-0.017
Heavy smoking	189,133	9,308	4.69	168,377	7,141	4.07	<0.001	-0.015	218,341	1,236	0.56	192,998	1,002	0.52	0.044	-0.003
Drinking habits																
Binge drinking	183,345	15,502	7.80	162,731	12,748	7.26	<0.001	-0.010	215,155	4,406	2.01	190,033	3,657	1.89	0.006	-0.004
Heavy drinking	189,922	8,925	4.49	167,567	7,912	4.51	0.764	0.000	217,494	2,067	0.94	191,696	1,994	1.03	0.004	0.004
Dietary habits																
Eating meals regularly	97,378	101,265	51.0	81,726	93,840	53.4	<0.001	0.025	91,546	127,268	58.2	78,131	115,224	59.6	<0.001	0.014
Eating a balanced diet	134,034	64,609	32.5	114,122	61,444	35.0	<0.001	0.026	132,504	86,310	39.4	113,713	79,642	41.2	<0.001	0.018
Eating a low-salt diet	154,572	44,071	22.2	137,288	38,278	21.8	0.005	-0.005	145,409	73,405	33.5	129,150	64,205	33.2	0.021	-0.004
Fitness habits																
Having an exercise habit	125,618	73,025	36.8	106,756	68,810	39.2	<0.001	0.025	147,670	71,144	32.5	125,969	67,386	34.9	<0.001	0.025
Mental health																
Those with SPD	186,314	7,032	3.64	165,101	6,380	3.72	0.183	0.002	202,286	9,928	4.68	179,208	8,918	4.74	0.358	0.001

ES, effect size; SPD, serious psychological distress. Those with missing data were excluded from each analysis. Due to the large sample size, statistical significance was set at *P* < 0.01 level.

Heavy smoking means >20 cigarettes per day.

Binge drinking (= heavy episodic drinking) means 60+ grams of alcohol on at least one occasion during past 30 days.

Heavy drinking means drinking an average of 60 grams or more per day.

Serious psychological distress means a total K6 score of 13 or more. K6 means the Kessler 6-item Psychological Distress Scale.

### Sleep duration and nonrestorative sleep

**Fig. 1:**
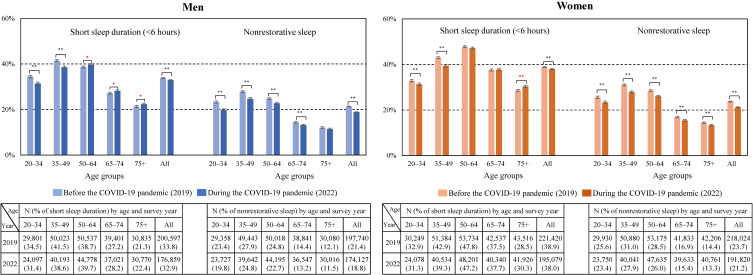
Prevalence of short sleep duration and nonrestorative sleep by gender and age before and during the COVID-19 pandemic. **P < 0.001, *P < 0.01. Red asterisks indicate a significant increase.

Regarding sleep duration, the prevalence of short sleep duration (<6 hours) was significantly lower in 2022 than in 2019 for both men and women: The prevalence among men was 33.8% in 2019 and 32.9% in 2022, and the prevalence among women was 38.9% in 2019 and 38.0% in 2022 (Fig. 1). By gender and age, there was a significant decrease among both men and women under the age of 49, but there was a significant increase for men aged 50 over and women aged 75 over.

Regarding NRS, the prevalence of NRS was significantly lower in 2022 than in 2019 for both men and women: The prevalence among men was 21.4% in 2019 and 18.8% in 2022, and the prevalence among women was 23.7% in 2019 and 21.2% in 2022. Moreover, the prevalence of NRS decreased significantly among men aged 74 years or younger and women in all age groups.

### Prevalence of NRS by sleep duration

**Fig. 2:**
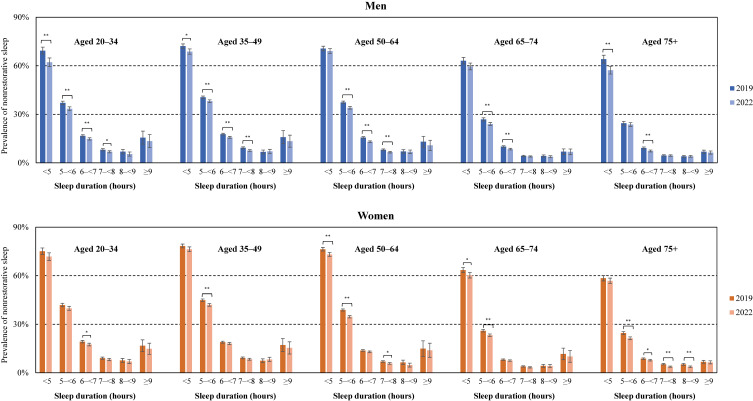
Prevalence of nonrestorative sleep by sleep duration in men and women, 2019 and 2022. **P < 0.001, *P < 0.01.

Sleep duration, for which the prevalence of people with NRS was significantly lower in 2022 than in 2019, differed by gender and age (Fig. 2). However, overall, the prevalence of NRS was significantly reduced in people with relatively short sleep duration. This analysis suggested that the decrease in the prevalence of NRS was due to a decrease in NRS in the group with short sleep duration.

### Adjusted prevalence of NRS according to survey year

Using a generalized estimating equation that assumes a Poisson distribution, we estimated the adjusted prevalence of people with NRS in each survey year, controlling for factors that affect NRS. In crude model, the difference in the adjusted prevalence of NRS in 2022 compared to the adjusted prevalence in 2019 was minus 2.53 pp (95% confidence interval [CI] minus 2.72 pp to minus 2.33 pp, *P* < 0.001), and this difference showed a significant decrease (Table 2). In Model 1, where the data were adjusted for gender, age, and sleep duration, the difference in NRS prevalence was minus 1.50 pp (95% CI minus 1.65 pp to minus 1.35 pp, *P* < 0.001), and the difference remained significant. In Model 2, where the data were additionally adjusted for socioeconomic status, illness under treatment, lifestyle behaviors, and mental health, the difference in NRS prevalence was smaller at minus 1.48 pp (95% CI minus 1.65 pp to minus 1.31 pp, *P* < 0.001), but a significant association in which the prevalence of NRS decreased in 2022 compared to 2019 was maintained.

**Table 2:** Prevalence of nonrestorative sleep according to survey year

**Model**	**Survey year**	**N**	**% NRS (95% CI)**	**Percent point (95% CI)**	***P*-value**
Crude	2019	415,764	22.6% (22.5% to 22.7%)	-2.53 pp (-2.72 pp to -2.33 pp)	<0.001
	2022	365,947	20.1% (19.9% to 20.2%)		

Model 1	2019	415,764	18.5% (18.2% to 18.8%)	-1.50 pp (-1.65 pp to -1.35 pp)	<0.001
	2022	365,947	17.0% (16.7% to 17.2%)		

Model 2	2019	415,764	21.3% (20.8% to 21.9%)	-1.48 pp (-1.65 pp to -1.31 pp)	<0.001
	2022	365,947	19.8% (19.3% to 20.4%)		

CI, confidence interval; NRS, nonrestorative sleep; pp, percent point.

Those with missing data on NRS (n = 10,746 in 2019, n = 9,631 in 2022) were excluded from these analyses.

Percent point is calculated by subtracting the 2019 percentage from the 2022 percentage.

The generalized estimating equations of the multivariable Poisson regression models were used as a method of statistical analysis.

Model 1: Adjusted for gender, age, and sleep duration. Model 2: Model 1 plus socioeconomic status, illness under treatment, lifestyle behaviors, and mental health. Socioeconomic status includes marital status, family size, housing tenure, equivalent household expenditures, education, and employment status. Lifestyle behaviors include smoking status, alcohol intake, and no. of dietary and fitness habits practiced.

Because the prevalence of NRS may vary according to age, gender, and employment status, we conducted additional analyses stratified by these factors. Regardless of age, gender, and employment status, the prevalence of NRS decreased significantly in 2022 compared to 2019 (Additional file 6).

### Crude prevalence of NRS between 2014 and 2021 based on the NDBODJ

**Fig. 3:**
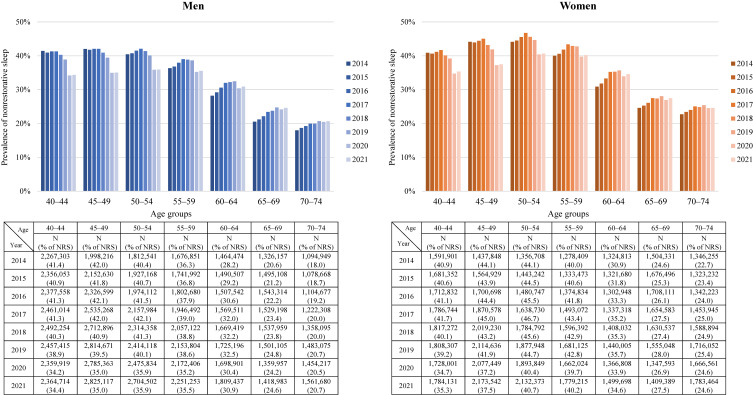
Prevalence of nonrestorative sleep based on the NDB Open Data Japan

To verify whether a significant improvement of NRS would be observed in the first and second years of the COVID-19 pandemic, we used the NDBODJ and examined the crude prevalence of NRS from 2014 to 2021. Compared to 2019, the crude prevalence of NRS was lower in 2020–2021 for both men and women of all ages, with a particularly large decline among working-age people aged 40 to 54 (Fig. 3).

## Discussion

Regarding Japanese people’s sleep symptoms during the COVID-19 pandemic, overall, both the prevalence of short sleep duration (i.e., sleep of less than 6 hours) and the prevalence of NRS decreased compared to before the pandemic. However, the decrease in short sleep duration was observed among men and women under the age of 49, but NRS improved regardless of gender and age. Furthermore, NRS significantly decreased independently of the prevalence of short sleep duration in 2022 compared to 2019. To our knowledge, this study is the first to demonstrate that Japanese NRS improved during the COVID-19 pandemic, even after considering potential confounders including sleep duration.

A meta-analysis examining the effects of the COVID-19 pandemic on sleep in healthy adults reported that sleep duration increased and sleep quality decreased [20]. Regarding sleep duration, in our study, the prevalence of short sleep duration decreased overall, which is consistent with previous research. The reason for the increase in sleep duration during the COVID-19 pandemic is that due to refraining from social activities and the spread of working from home, people may have had more time to sleep as they no longer needed to go out and were free to manage their own time [20]. On the other hand, this study showed a decrease in the prevalence of NRS, i.e., an improvement in sleep quality, which was inconsistent with previous studies. Previous research has shown that during the COVID-19 pandemic, sleep was negatively affected by isolation, fear of being infected, worries about employment and money, reduced physical activity, decreased exposure to daylight, and increased use of ICT devices [12, 21, 22]. On the other hand, it has been reported that the impact of the COVID-19 pandemic on perceived sleep quality is dependent on pre-pandemic sleep quality [23]: Pre-pandemic good sleepers were more likely to experience worse sleep during the lockdown measures, while pre-pandemic poor sleepers tended to have a meaningful improvement in sleep quality. Previous studies have reported that reduced social time pressure, as reflected by increased frequency of work-at-home and online classes, increased sleep duration and reduced the difference between weekday and weekend sleep duration (i.e., social jetlag or social sleep restriction) [24–27]. Changes in sleep duration and social sleep restriction have been reported to be more pronounced in those who had shorter sleep duration before the COVID-19 pandemic [27], young adults [26], and those living in a metropolitan area [25]. The improvement in NRS during the COVID-19 pandemic in this study may have been brought about by the alleviation of social time pressures. However, this explanation is only possible for workers. As shown in Additional file 6, the decline in NRS was observed not only among workers but also among non-working people. Therefore, other explanations need to be considered. A longitudinal study of young people in the United States reported that the pandemic caused only slight changes in young people’s overall mental health, but young people entering the pandemic with pre-pandemic mental health problems showed significant improvements across all outcomes [28]: One possible explanation is that the social restrictions associated with the pandemic meant a withdrawal from stressful social environments, which may have had a positive effect on the mental health of young people with existing mental health problems. The results of this study suggest that social restrictions due to the COVID-19 pandemic are associated with improved sleep quality in Japanese people. Previous studies in Japan have pointed out that obligatory social interactions may have a negative effect on mental health [29, 30]: Obligatory social participation may impose a psychological burden on participants [31]. In addition, when social participation is obligatory, participants do not build strong connections with other people and are not cooperative, so such social interactions may not have a stress-relieving effect [32]. Obligatory social interactions for Japanese people might include Parents and Teachers Association (PTA) and community association activities, work-related drinking parties, and socializing with relatives [29–31]. Social restrictions due to the COVID-19 pandemic may have freed Japanese people from obligatory social interactions, leading to a decrease in people with NRS.

This study was conducted in the third year of the COVID-19 pandemic. Because many previous studies were conducted in the first year of the COVID-19 pandemic [12, 13, 21, 22, 26], the impact on sleep quality may have differed between this study and the previous studies. For example, it is possible that people had adapted to the COVID-19 pandemic over the three-year period [33]. Therefore, using open data compiled from specific health checkup information in Japan, we examined the prevalence of NRS in the first and second years of the COVID-19 pandemic, and found that the prevalence of NRS had decreased. In other words, it is difficult to explain the results of this study as being due to adaptation to the COVID-19 pandemic.

As for why the prevalence of NRS decreased in those with short sleep duration, establishing a consistent sleep schedule is essential to prevent NRS [34]. The Global Chrono Corona Survey of adults in 40 countries reported that during the COVID-19 social restrictions, most people’s sleep and wake times on weekdays were closer to those on weekends before the social restrictions, promoting a reduction in the use of alarm clocks [35]. This consistency strengthened natural circadian rhythms, which may have led to more restorative sleep, even for short sleep duration, leading to a reduction in the proportion of people with NRS.

This study suggested that in the short sleep group, there were significant differences in the proportion of NRS for men aged 20–49 and for women aged 50–74 between 2019 and 2022 (Fig. 2). Therefore, we considered possible explanations for age and gender differences in these significant differences. First, regarding age, because older people are at higher risk of developing severe symptoms of COVID-19 [36], they are more anxious and fearful of COVID-19 than younger people, which may have a negative impact on their mental health and reduce the quality of their sleep. Second, regarding gender differences, it has been reported that in Japan, people have spent more time at home due to the COVID-19 pandemic, and women have spent more time doing housework [37]. This increased burden of housework may have made it difficult for women to maintain a consistent sleep rhythm, and they may not have been able to improve their sleep quality as much as men. Third, regarding the employment and labor situation, the spread of COVID-19 has had negative effects, such as a significant decline in the number of employed people and a reduction in working hours and wages, but these negative effects have been reported to be more prevalent among non-regular workers than among regular workers [38]. On the other hand, it has been reported that the adoption rate of telecommuting is higher among regular workers than among non-regular workers [38]. Given that a higher proportion of men are in full-time employment than women [39], female workers may be more vulnerable to pandemic-related negative impacts, while male workers may enjoy the benefit associated with the pandemic. Finally, regarding interpersonal interactions, middle-aged and young-old women play a central role in interacting with relatives and neighbors. The social constraints associated with the pandemic allowed middle-aged and young-old women to escape from stressful and obligatory social interactions, which may have had a positive impact on the quality of their sleep. In addition, parents with children have had to spend more time caring for their children due to school closures and other factors, but in Japan the burden of childcare tends to fall more on mothers than fathers [37]. In summary, the effects of pandemic-related social restrictions may be such that, among women, younger generations are more likely to be negatively affected and middle-aged and young-old people more likely to benefit. Meanwhile, among men, those in their prime working years may be more likely to benefit. The above is consistent as an explanation for the results observed in Fig. 2.

This study has several strengths. First, because this study used large-scale national survey data, we were able to analyze participants aged 20 years and older by gender and age. Although gender and age differences have been pointed out regarding the risk of severe COVID-19 and the health effects of home confinement during the COVID-19 pandemic [36, 40], previous studies [13, 23, 41] were unable to perform stratified analyses by gender and age. Second, this study was based on a self-administered questionnaire. Previous studies during the COVID-19 pandemic conducted online surveys [11, 14, 23–25, 33, 35, 41]. Due to concerns about selection bias among the participants of the online survey, there were limitations to the generalizability of research results to the general population. Third, because the CSLC included wide-ranging variables such as sleep duration, mental health, lifestyle behaviors, and socioeconomic status, we were able to adjust for essential confounding factors.

This study has several limitations. First, because the CSLC was a cross-sectional survey, we failed to assess changes in sleep duration and NRS within individuals before and during the COVID-19 pandemic. Future research should conduct prospective cohort studies to assess within-individual changes. Second, the sleep variables were self-reported without the objective sleep measures. It is known that there is often a discrepancy between self-reported sleep duration and objectively measured sleep duration [42]. Future research needs to evaluate the relationship between objective sleep duration and NRS. Third, the valid response rate for this study was 72.1% in 2019, but dropped to 68.0% in 2022. It is speculated that those who did not respond to the 2022 survey compared to the 2019 survey were younger, but other characteristics are unknown. One possible characteristic is that, because the CSLC uses a survey method in which the investigators distribute and collect questionnaires, those in poor health may have avoided contact with others to prevent infection and therefore did not submit the questionnaire, compared to those in good health: In 2022, when the response rate was low, there may have been more healthy respondents than in 2019, when the response rate was high. Therefore, it should be noted that the conclusion that the NRS improved in 2022 compared to 2019 may be overestimated. Fourth, another possible explanation for the lower response rate in 2022 may be the difference in survey method. Mail collection was used as an acceptable method when “surveyors cannot meet the respondents in person even after repeated visits,” but there was no specific guideline for the number of visits. In the 2022 survey, as a special measure to prevent the spread of COVID-19, the guideline for the number of visits by surveyors was set at three times [43]. It was anticipated that this change in survey method in 2022 would increase the likelihood that workers, who commute to their workplaces and are often absent from home, would return their completed questionnaires by mail. However, this may have led to less questionnaires being returned by working-age people, which is supported by the fact that the response rate of younger people, i.e., the working-age generation, was lower in the 2022 survey than in the 2019 survey. Fifth, in this study, the NRS was assessed using a single self-reported question with 4-point Likert-scale, which has not been verified for validity and reliability. Self-administered questionnaires for the NRS with established validity and reliability include a single question with 5-point Likert scale [44], the 12-item Nonrestorative Sleep Scale [45], and the 9-item short version [46], but neither have been developed in Japanese. Future studies should confirm the findings of the current study using the Japanese version of the NRS assessment, whose validity and reliability have been established.

### Conclusion with implications

This study provides basic information on sleep symptoms in Japanese people and contributes to the existing literature investigating sleep quality during the COVID-19 pandemic. While there are many reports that sleep quality has decreased during the COVID-19 pandemic, our results suggest that NRS in the general Japanese population has improved during the COVID-19 pandemic. We need to monitor whether the declines in the NRS prevalence acquired during the COVID-19 pandemic persist after the pandemic or return to pre-pandemic conditions. Additionally, based on our findings, there is scope to consider continuing measures taken during the COVID-19 pandemic, such as encouraging teleworking.

## Abbreviations

CI: confidence interval
CSLC: Comprehensive Survey of Living Condition
K6: Kessler 6-item Psychological Distress Scale
NDB: National Database of Health Insurance Claims and Specific Health Checkups of Japan
NDBODJ: NDB Open Data Japan
NRS: nonrestorative sleep
pp: percent point
SES: socioeconomic status
SHC: specific health checkups

## Supplementary information

**Additional file 1.** Estimated proportion of valid responses by gender and age for each survey year. **Additional file 2.** Selection of study participants. **Additional file 3.** The reasons for excluding certain participants from the study. **Additional file 4.** Detailed explanations of covariates. **Additional file 5.** Distribution of responses to covariates among study participants. **Additional file 6.** Subgroup analysis for nonrestorative sleep based on fully adjusted model.

## Declarations

### Availability of data and materials

Data are available from the Ministry of Health, Labour and Welfare, Japan (https://www.mhlw.go.jp/stf/toukei/goriyou/chousahyo.html) for researchers who obtain approval to use questionnaire information in accordance with the Statistics Act of Japan.
