# Supplementary material for: Prevalence of nonrestorative sleep before and during the COVID-19 pandemic: based on a nationwide cross-sectional survey among Japanese in 2019 and 2022
Source: Environ Health Prev Med. 2025 Jan 25;30:6. doi: 10.1265/ehpm.24-00197 (PMC11790402; doi:10.1265/ehpm.24-00197)
Supplement: Supplementary file 1 — Additional file 1. Estimated proportion of valid responses by gender and age for each survey year. Additional file 2. Selection of study participants. Additional file 3. The reasons for excluding certain participants from the study. Additional file 4. Detailed explanations of covariates. Additional file 5. Distribution of responses to covariates among study participants. Additional file 6. Subgroup analysis for nonrestorative sleep based on fully adjusted model. [file ehpm-30-006-s001.docx]

**Additional file 1.** Estimated proportion of valid responses by gender and age for each survey year

| Age group | | Survey year: 2019 | | |  | Survey year: 2022 | | | Percent point |
| --- | --- | --- | --- | --- | --- | --- | --- | --- | --- |
|  |  | Estimated number of distributions | Number of valid responses | Estimated proportion of valid responses |  | Estimated number of distributions | Number of valid responses | Estimated proportion of valid responses |  |
| **Men** | |  |  |  |  |  |  |  |  |
|  | <20 | 62,720 | 47,161 | 75.2% |  | 56,356 | 37,191 | 66.0% | -9.2 pp |
|  | 20-34 | 58,000 | 31,263 | 53.9% |  | 53,854 | 26,334 | 48.9% | -5.0 pp |
|  | 35-49 | 76,844 | 51,906 | 67.5% |  | 68,602 | 43,051 | 62.8% | -4.8 pp |
|  | 50-64 | 69,156 | 52,581 | 76.0% |  | 68,502 | 47,783 | 69.8% | -6.3 pp |
|  | 65-74 | 48,416 | 41,267 | 85.2% |  | 44,311 | 38,982 | 88.0% | 2.7 pp |
|  | 75+ | 42,373 | 33,638 | 79.4% |  | 42,042 | 33,552 | 79.8% | 0.4 pp |
|  | All ages | 357,509 | 257,816 | 72.1% |  | 333,666 | 226,893 | 68.0% | -4.1 pp |
| **Women** | |  |  |  |  |  |  |  |  |
|  | <20 | 60,811 | 44,366 | 73.0% |  | 54,677 | 35,020 | 64.0% | -8.9 pp |
|  | 20-34 | 55,855 | 31,614 | 56.6% |  | 52,195 | 26,220 | 50.2% | -6.4 pp |
|  | 35-49 | 76,330 | 53,051 | 69.5% |  | 67,986 | 43,434 | 63.9% | -5.6 pp |
|  | 50-64 | 70,760 | 55,630 | 78.6% |  | 69,929 | 51,016 | 73.0% | -5.7 pp |
|  | 65-74 | 53,896 | 44,455 | 82.5% |  | 49,317 | 42,580 | 86.3% | 3.9 pp |
|  | 75+ | 66,458 | 47,855 | 72.0% |  | 65,612 | 46,337 | 70.6% | -1.4 pp |
|  | All ages | 384,110 | 276,971 | 72.1% |  | 359,716 | 244,607 | 68.0% | -4.1 pp |

pp, percent point.

Percent point is calculated by subtracting the 2019 percentage from the 2022 percentage.

This estimate assumes that the number of household members surveyed corresponds to the Japanese population by gender and age.

**Additional file 2.** Selection of study participants

|  |  | Survey year | | Total |
| --- | --- | --- | --- | --- |
|  |  | 2019 | 2022 |  |
| No. of households to which questionnaires were distributed | | 301,334 | 299,772 | 601,106 |
| No. of households that returned their questionnaire | | 218,335 | 205,063 | 423,398 |
|  | Response rate | 72.5% | 68.4% | 70.4% |
| No. of households with valid response | | 217,197 | 203,819 | 421,016 |
|  | Valid response rate | 72.1% | 68.0% | 70.0% |
| No. of persons with valid response | | 535,619 | 472,042 | 1,007,661 |
| No. of persons excluded from analysis | | 119,855 | 106,095 | 225,950 |
|  | Aged <20 | 91,527 | 72,211 | 163,738 |
|  | Hospitalized or nursing home admission | 7,399 | 6,311 | 13,710 |
|  | Age unknown | 832 | 542 | 1,374 |
|  | Hospitalization/admission status unknown | 9,351 | 17,400 | 26,751 |
| No. of persons analyzed | | 426,510 | 375,578 | 802,088 |

**Additional file 3.** The reasons for excluding certain participants from the study

We excluded from the study participants those under the age of 20, hospitalized people, those with nursing home admission, and whose age or hospitalization/admission status was unknown. The reasons for excluding certain participants from the study were as follows: First, people under the age of 20 were exempt from answering questions about their smoking and drinking habits because smoking and drinking are prohibited by law for people under 20 years of age in Japan. Second, data on sleep and lifestyle habits were not available for hospitalized or institutionalized individuals because they were exempt from answering health-related questions. Third, because this study was analyzed by age group, people whose age was unknown were excluded from the study participants. Finally, those whose hospitalization or admission status was unknown were excluded from the study participants because they were classified as missing for each response and did not have information on any of the variables.

**Additional file 4.** Detailed explanations of covariates

a) Explanation of the chosen cutoff values ​​for age

The age range for what age is considered older people varies depending on the era and region, but the general definition is 65 years and older [1]. In Japan, the Act on Assurance of Medical Care for Elderly People defines "older people" as 65 years and older, and divides them into those aged 65 to 74 (the “young-old”) and those aged 75 and older (the “old-old”) [2]. In Health Japan 21, promoted by the Ministry of Health, Labour and Welfare, 15 to 24 years old is youth, 25 to 44 years old is prime age, 45 to 64 years old is middle age, and 65 years and older is senior [3]. In this study, the cutoff value selected for age was basically 15 years, except for the young-old, which was divided into 10 years. Although this division is not an equal 15-year division, it allows for age classification into young, prime-aged, middle-aged, young-old, and old-old, and is convenient for gender and age-specific analysis as there are no groups with extremely small numbers of people, making it a better definition of age for this study.

b) Details of equivalent household expenditures (EHE)

EHE (Japanese thousand yen per month) were divided into five categories by quintiles: first quintile (<89), second quintile (89–115), third quintile (116–144), fourth quintile (145–184), and fifth quintile (≥184).

c) Details of employment status

Regarding employment status, it has been reported that the impact of the COVID-19 recession (e.g., layoffs and economic anxiety) was greater on non-regular workers and small businesses [4]. Therefore, employment status was classified into the following eight groups according to employment contract and business size: full-time workers at companies with 300 or more employees (hereinafter referred to as large companies), full-time workers at companies with 30 to 299 employees (hereinafter referred to as medium companies), full-time workers at companies with fewer than 30 employees (hereinafter referred to as small companies), civil servants, non-regular employees, self-employed, other (e.g. company or organization officials, full-time employees of unknown company size), and non-working.

d) Details of illness under treatment

Regarding illness under treatment, first, the Comprehensive Survey of Living Conditions asks whether or not there is an illness currently being treated. Next, those who answered that they are currently receiving treatment are asked to select the corresponding number from the following illnesses: diabetes; obesity; dyslipidemia; thyroid diseases; mental and neurological diseases, including depression, dementia, and Parkinson’s disease; vision disorders; hearing disorders; cardiovascular diseases, including hypertension, stroke, and coronary artery diseases; respiratory diseases, including chronic obstructive pulmonary disease, asthma, and allergic rhinitis; diseases of digestive organs, including the stomach, duodenum, liver, and gall bladder; dental diseases; skin disorders, including atopic dermatitis; muscular-skeletal disorders, including gout, rheumatoid arthritis, arthropathy, backache, and osteoporosis; urinary and genital diseases, including renal diseases, prostatic hyperplasia, and menopausal disorders; injuries, including bone fractures and burns; hematological diseases; and cancer. If each illness were evaluated individually, there would be overlap in the "unknown medical history" group. Therefore, in this study, illness under treatment was defined as persons currently being treated for at least one of the aforementioned illnesses.

e) Details of alcohol intake (amount of pure alcohol consumed per day)

Regarding alcohol intake, first, the Comprehensive Survey of Living Conditions asks respondents how many days a week they drink alcohol. Next, those who answer that they drink alcohol at least once a month are asked to convert the amount of alcohol consumed per day into sake. The amount of pure alcohol consumed per day for each respondent was calculated by assuming that one cup (180 ml) of sake is 20 g of pure alcohol. The guideline for healthy drinking [5] uses the amount of pure alcohol consumed per day to define the amount of alcohol that increases the risk of lifestyle-related diseases as 40 grams or more for men and 20 grams or more for women, and heavy drinking as 60 grams or more. Therefore, in this study, participants were classified into six groups based on the amount of pure alcohol consumed per day: non-drinkers, social drinkers, habitual-low (<20g), habitual-medium (20–39g), habitual-high (40–59g), habitual-heavy (≥60g).

References:

1. Tanaka K, Osuka Y. Reflecting on the age categories of older adults. Journal of gerontological nursing and caring research 2021;12:15–19. (Japanese).
2. e-GOV Law Search. Act on Assurance of Medical Care for older adults. <https://elaws.e-gov.go.jp/document?lawid=357AC0000000080_20221209_504AC0000000096>. Accessed 15 Dec 2024. (Japanese).
3. Ministry of Health, Labour and Welfare. Health Japan 21. <https://www.mhlw.go.jp/www1/topics/kenko21_11/top.html>. Accessed 15 Dec 2024. (Japanese).
4. Ministry of Health, Labour and Welfare, Japan. Analysis of the Labor Economy, 2021: The impact of COVID-19 on employment and labor. <https://www.mhlw.go.jp/stf/wp/hakusyo/roudou/20/20-1.html>. Accessed 15 Dec 2024. (Japanese).
5. Ministry of Health, Labour and Welfare, Japan. <https://www.mhlw.go.jp/stf/newpage_38541.html>. Accessed 15 Dec 2024. (Japanese).

**Additional file 5.** Distribution of responses to covariates among study participants

|  |  | All study participants  (N = 802,088) |  | Participants for  fully adjusted model  (N = 781,711) |
| --- | --- | --- | --- | --- |
|  |  | N (%) |  | N (%) |
| Survey year | |  |  |  |
|  | 2019 | 426,510 (53.2) |  | 415,764 (53.2) |
|  | 2022 | 375,578 (46.8) |  | 365,947 (46.8) |
| Gender | |  |  |  |
|  | Men | 381,426 (47.6) |  | 371,867 (47.6) |
|  | Women | 420,662 (52.4) |  | 409,844 (52.4) |
| Age |  |  |  |  |
|  | Aged 20–34 | 109,214 (13.6) |  | 106,765 (13.7) |
|  | Aged 35–49 | 183,447 (22.9) |  | 180,006 (23.0) |
|  | Aged 50–64 | 198,666 (24.8) |  | 195,023 (24.9) |
|  | Aged 65–74 | 161,004 (20.1) |  | 156,854 (20.1) |
|  | Aged 75+ | 149,757 (18.7) |  | 143,063 (18.3) |
| Marital status | |  |  |  |
|  | Married | 521,915 (65.1) |  | 510,053 (65.2) |
|  | Never-married | 153,648 (19.2) |  | 149,433 (19.1) |
|  | Widowed/divorced | 126,525 (15.8) |  | 122,225 (15.6) |
| Family size | |  |  |  |
|  | Five or more | 97,776 (12.2) |  | 95,257 (12.2) |
|  | Four | 134,978 (16.8) |  | 132,133 (16.9) |
|  | Three | 203,741 (25.4) |  | 198,730 (25.4) |
|  | Two | 254,837 (31.8) |  | 248,103 (31.7) |
|  | One (living alone) | 110,756 (13.8) |  | 107,488 (13.8) |
| Housing tenure | |  |  |  |
|  | Owner-occupied | 635,486 (79.2) |  | 619,599 (79.3) |
|  | Privately rented | 98,115 (12.2) |  | 96,023 (12.3) |
|  | Provided housing | 13,597 (1.7) |  | 13,390 (1.7) |
|  | Publicly subsidized | 23,033 (2.9) |  | 22,343 (2.9) |
|  | Rented rooms | 31,857 (4.0) |  | 30,356 (3.9) |

**Additional file 5.** Continued

|  |  | All study participants  (N = 802,088) |  | Participants for  full adjusted model  (N = 781,711) |
| --- | --- | --- | --- | --- |
|  |  | N (%) |  | N (%) |
| Equivalent household expenditures (Japanese thousand yen per month) | | | | |
|  | 5th quintile (>184) | 152,952 (19.1) |  | 150,048 (19.2) |
|  | 4th quintile (145–184) | 145,466 (18.1) |  | 142,518 (18.2) |
|  | 3rd quintile (116–144) | 152,390 (19.0) |  | 149,029 (19.1) |
|  | 2nd quintile (89–115) | 163,385 (20.4) |  | 159,382 (20.4) |
|  | 1st quintile (<89) | 150,274 (18.7) |  | 145,696 (18.6) |
|  | Missing | 37,621 (4.7) |  | 35,038 (4.5) |
| Education (years of schooling) | |  |  |  |
|  | >15 years | 160,696 (20.0) |  | 158,512 (20.3) |
|  | 13–15 years | 133,760 (16.7) |  | 131,604 (16.8) |
|  | 10–12 years | 293,453 (36.6) |  | 287,336 (36.8) |
|  | <10 years | 87,352 (10.9) |  | 84,234 (10.8) |
|  | Missing | 126,827 (15.8) |  | 120,025 (15.4) |
| Employment status | |  |  |  |
|  | Full-time workers at large companies | 108,190 (13.5) |  | 106,708 (13.7) |
|  | Full-time workers at medium companies | 104,432 (13.0) |  | 102,726 (13.1) |
|  | Full-time workers at small companies | 76,420 (9.5) |  | 75,079 (9.6) |
|  | Civil servants | 23,684 (3.0) |  | 23,387 (3.0) |
|  | Non-regular employees | 77,839 (9.7) |  | 76,212 (9.7) |
|  | Self-employed | 51,853 (6.5) |  | 50,636 (6.5) |
|  | Other | 44,338 (5.5) |  | 43,009 (5.5) |
|  | Non-working | 284,724 (35.5) |  | 276,056 (35.3) |
|  | Missing | 30,608 (3.8) |  | 27,898 (3.6) |
| Illness under treatment | |  |  |  |
|  | Absent | 397,634 (49.6) |  | 388,795 (49.7) |
|  | Present | 398,806 (49.7) |  | 389,038 (49.8) |
|  | Missing | 5,648 (0.7) |  | 3,878 (0.5) |

**Additional file 5.** Continued

|  |  | All study participants  (N = 802,088) |  | Participants for fully adjusted  model (N = 781,711) |
| --- | --- | --- | --- | --- |
|  |  | N (%) |  | N (%) |
| Smoking status (cigarettes per day) | | | | |
|  | Non-smokers | 604,006 (75.3) |  | 591,558 (75.7) |
|  | Ex-smokers | 44,720 (5.6) |  | 44,183 (5.7) |
|  | Current-light (1–10) | 49,005 (6.1) |  | 47,983 (6.1) |
|  | Current-moderate (11–20) | 71,118 (8.9) |  | 69,908 (8.9) |
|  | Current-heavy (21+) | 18,687 (2.3) |  | 18,348 (2.3) |
|  | Missing | 14,552 (1.8) |  | 9,731 (1.2) |
| Alcohol intake (average amount of alcohol per day) | | | | |
|  | Non-drinkers | 312,405 (38.9) |  | 304,743 (39.0) |
|  | Social drinkers | 151,447 (18.9) |  | 148,603 (19.0) |
|  | Habitual-low (<20g) | 185,347 (23.1) |  | 182,481 (23.3) |
|  | Habitual-medium (20–39g) | 83,747 (10.4) |  | 82,417 (10.5) |
|  | Habitual-high (40–59g) | 33,733 (4.2) |  | 33,219 (4.2) |
|  | Habitual-heavy (≥60g) | 20,898 (2.6) |  | 20,574 (2.6) |
|  | Missing | 14,511 (1.8) |  | 9,674 (1.2) |
| The number of dietary and fitness habits practiced | | | | |
|  | 0 | 209,274 (26.1) |  | 205,022 (26.2) |
|  | 1 | 204,552 (25.5) |  | 200,225 (25.6) |
|  | 2 | 169,092 (21.1) |  | 166,010 (21.2) |
|  | 3 | 126,650 (15.8) |  | 124,423 (15.9) |
|  | 4 | 76,810 (9.6) |  | 75,485 (9.7) |
|  | Missing | 15,710 (2.0) |  | 10,546 (1.3) |
| Psychological distress (K6 score) | | | | |
|  | None (0–4) | 553,943 (69.1) |  | 545,318 (69.8) |
|  | Mild (5–12) | 178,966 (22.3) |  | 176,073 (22.5) |
|  | Serious (13+) | 32,258 (4.0) |  | 31,821 (4.1) |
|  | Missing | 36,921 (4.6) |  | 28,499 (3.6) |
| Sleep duration | |  |  |  |
|  | <5hr | 60,985 (7.6) |  | 60,120 (7.7) |
|  | 5–6hr | 225,198 (28.1) |  | 221,740 (28.4) |
|  | 6–7hr | 264,141 (32.9) |  | 260,447 (33.3) |
|  | 7–8hr | 175,391 (21.9) |  | 171,677 (22.0) |
|  | >8hr | 68,240 (8.5) |  | 66,154 (8.5) |
|  | Missing | 8,133 (1.0) |  | 1,573 (0.2) |

**Additional file 6**. Subgroup analysis for nonrestorative sleep based on fully adjusted model

| Subgroups | | Survey year | N | Adjusted % (95% CI) | Percent point (95% CI) | *P*-value |
| --- | --- | --- | --- | --- | --- | --- |
| **By age^a^** | |  |  |  |  |  |
|  | Aged 20–34 | 2019 | 59,288 | 24.2% (22.5% to 26.0%) | -1.34 pp (-1.81 pp to -0.86 pp) | <0.001 |
|  |  | 2022 | 47,477 | 22.9% (21.2% to 24.6%) |  |  |
|  | Aged 35–49 | 2019 | 100,323 | 23.6% (22.3% to 24.9%) | -1.25 pp (-1.56 pp to -0.94 pp) | <0.001 |
|  |  | 2022 | 79,683 | 22.3% (21.1% to 23.6%) |  |  |
|  | Aged 50–64 | 2019 | 103,193 | 23.9% (22.8% to 25.1%) | -1.91 pp (-2.22 pp to -1.59 pp) | <0.001 |
|  |  | 2022 | 91,830 | 22.0% (21.0% to 23.2%) |  |  |
|  | Aged 65–74 | 2019 | 80,674 | 19.1% (17.8% to 20.6%) | -1.50 pp (-1.90 pp to -1.09 pp) | < 0.001 |
|  |  | 2022 | 76,180 | 17.6% (16.4% to 19.0%) |  |  |
|  | Aged 75+ | 2019 | 72,286 | 14.2% (12.8% to 15.8%) | -1.42 pp (-1.77 pp to -1.07 pp) | <0.001 |
|  |  | 2022 | 70,777 | 12.8% (11.5% to 14.2%) |  |  |
| **By gender^b^** | |  |  |  |  |  |
|  | Men | 2019 | 197,740 | 21.2% (20.4% to 22.0%) | -1.53 pp (-1.77 pp to -1.29 pp) | <0.001 |
|  |  | 2022 | 174,127 | 19.7% (18.9% to 20.5%) |  |  |
|  | Women | 2019 | 218,024 | 21.3% (20.6% to 22.1%) | -1.42 pp (-1.63 pp to -1.21 pp) | <0.001 |
|  |  | 2022 | 191,820 | 19.9% (1.92% to 20.6%) |  |  |
| **By employment status^c^** | | | | | | |
|  | Full-time large | 2019 | 56,381 | 20.5% (18.7% to 22.4%) | -1.14 pp (-1.50 pp to -0.78 pp) | <0.001 |
|  |  | 2022 | 50,327 | 19.3% (17.7% to 21.1%) |  |  |
|  | Full-time medium | 2019 | 55,755 | 22.5% (20.8% to 24.3%) | -1.37 pp (-1.78 pp to -0.96 pp) | <0.001 |
|  |  | 2022 | 46,971 | 21.1% (19.5% to 22.9%) |  |  |
|  | Full-time small | 2019 | 40,929 | 20.4 % (18.6% to 22.5%) | -1.34 pp (-1.81 pp to -0.87 pp) | <0.001 |
|  |  | 2022 | 34,150 | 19.1% (17.4% to 21.0%) |  |  |
|  | Civil servants | 2019 | 12,483 | 22.4% (17.7% to 28.3%) | -1.42 pp (-2.26 pp to -0.57 pp) | 0.001 |
|  |  | 2022 | 10,904 | 21.0% (16.6% to 26.6%) |  |  |
|  | Non-regular | 2019 | 41,907 | 20.8% (19.2% to 22.6%) | -1.15 pp (-1.64 pp to -0.65 pp) | <0.001 |
|  |  | 2022 | 34,305 | 19.7% (18.1% to 21.3%) |  |  |
|  | Self employed | 2019 | 27,228 | 18.4% (16.2% to 20.8%) | -1.13 pp (-1.73 pp to -0.54 pp) | <0.001 |
|  |  | 2022 | 23,408 | 17.2% (16.2% to 20.8%) |  |  |
|  | Non-working | 2019 | 145,427 | 18.7% (17.8% to 19.6%) | -1.75 pp (-2.03 pp to -1.48 pp) | <0.001 |
|  |  | 2022 | 130,629 | 16.9% (16.1% to 17.8%) |  |  |

CI, confidence interval; pp, percent point. Percent point is calculated by subtracting the 2019 percentage from the 2022 percentage. Potential confounders include gender, age, marital status, family size, housing tenure, equivalent household expenditures, education, employment status, illness under treatment, lifestyle behaviors, mental health, and sleep duration. Lifestyle behaviors include smoking status, alcohol intake, and the number of dietary and fitness habits practiced. ^a^Adjusted for potential confounders. ^b^Adjusted for potential confounders except for gender. ^c^Adjusted for potential confounders except for employment status.
